# Measurement Method of Femoral Anteversion During Surgery for Trochanteric Fractures

**DOI:** 10.7759/cureus.72532

**Published:** 2024-10-28

**Authors:** Yo Kinami, Masahiro Horita, Kazuo Fujiwara

**Affiliations:** 1 Department of Orthopedic Surgery, Okayama City Hospital, Okayama, JPN; 2 Department of Orthopedic Surgery, Okayama University Graduate School of Medicine, Dentistry and Pharmaceutical Sciences, Okayama, JPN

**Keywords:** bland-altman plot, femoral anteversion, femoral rotation, intraoperative anteversion, lateral hip view, tabletop plane, trochanteric fractures

## Abstract

The acceptable range of bilateral differences in the femoral rotation is ≤15°, as a difference in femoral rotation over 15° may cause functional disturbance. However, femoral trochanteric fractures do not have clear indicators of rotation during surgery. This study aimed to verify the accuracy of this novel method for measuring femoral anteversion during trochanteric fracture surgery.

From August 2022 to August 2024, this study prospectively included patients with femoral trochanteric fractures treated using a cephalo-medullary nail with a lag-screw neck-shaft angle of 125°. During surgery, direct intraoperative anteversion (DIAV) was measured using the hip post axis in the lateral hip image based on a tabletop plane parallel to the floor. DIAV was then converted to corrected intraoperative anteversion (CIAV) using a quick chart derived from a graph of the axial projection. The accuracy was analyzed by comparing CIAV with computed tomography anteversion (CTAV) measurements obtained after surgery.

One hundred patients (25 male, 75 female) with a mean age of 86.7 years (range: 67-104 years) were included in this study. According to the Orthopaedic Trauma Association classification, there were 66 patients classified as A1, 23 as A2, and 11 as A3. The mean DIAV and CIAV were 8.2°±8.2° and 9.6°±9.7°, respectively, while the mean CTAV was 10.3°±10.4°. The median difference between CIAV and CTAV was 3° (range: 0°-9°), with 84 patients exhibiting differences of ≤5°. No significant differences were found between CIAV and CTAV (p = 0.054), whereas DIAV was significantly lower than CTAV (p < 0.001). CIAV and CTAV were strongly correlated (r = 0.936, p < 0.001). The Bland-Altman plot between CIAV and CTAV revealed that 98 patients were within the limits of agreement, and the plot distribution showed no trend.

The measurement method for assessing femoral anteversion using the hip post axis on the lateral hip image based on a tabletop plane parallel to the floor during surgery demonstrated sufficient accuracy for indicating anteversion in femoral trochanteric fractures.

## Introduction

The acceptable range of bilateral differences in the femoral rotation is ≤15°, as a difference in the femoral rotation greater than 15° may cause functional disturbance [1-7]. Diaphyseal fractures can be reduced using the trochanteric contour as a reference for rotation [8,9], whereas trochanteric fractures lack a clear indicator of rotation. In previous reports, the rate of malrotation of >15° in trochanteric fractures ranged from 25.7% to 40.0% [10-13].

In our previous study [14], measuring the lag screw angle with an iPhone (Apple Inc., CA, USA) during surgery based on a reference position allowed us to estimate lag screw anteversion for postoperative computed tomography (CT). This method serves as an indicator of anteversion during trochanteric fracture surgery. In the next step, we attempted to measure femoral anteversion in trochanteric fractures using this method during surgery; however, the trial was abandoned because of the lack of accuracy in measuring the angle between the lag screw guide pin and neck axes. In contrast, during the trial study, the method of directly measuring femoral anteversion using the hip post axis was recognized.

This study aimed to verify the accuracy of a novel method for measuring femoral anteversion during trochanteric fracture surgery.

## Technical report

Patients and study design

This prospective study was conducted at a single-level II trauma center in accordance with the Declaration of Helsinki. This study was approved by the Research Ethics Committee of our hospital. Informed consent was obtained via an opt-out option on the hospital website.

From August 2022 to August 2024, all patients with femoral trochanteric fractures (Orthopaedic Trauma Association classification: 31A1-3 [15]) treated using a cephalo-medullary nail with a lag screw neck-shaft angle of 125° were entered in this study. IPT-EF nails (HOMS, Nagano, Japan) were used for all patients. The protocol for measuring femoral anteversion was formulated in two steps: “determining the reference position/image before surgery” and “measuring anteversion during surgery.”

Determining the reference position/image before surgery

**Figure 1:**
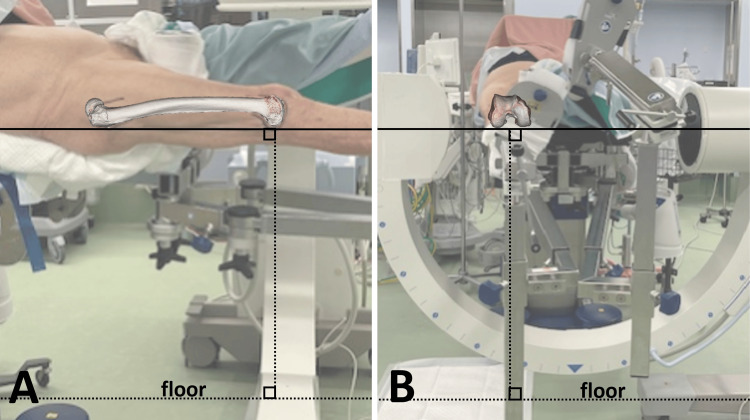
Reference position The tabletop plane was positioned parallel to the floor in the scissors position, with the lower limb pulled on the traction table (A/B). The tabletop plane was defined as a three-point plane based on the posterior edge of the greater trochanter and both posterior condyles. Alignment parallel to the floor in the sagittal direction (A) was established via palpation and visual inspection. The alignment parallel to the floor in the axial direction (B) was determined using a horizontal lateral knee view (Figure 2A).

**Figure 2:**
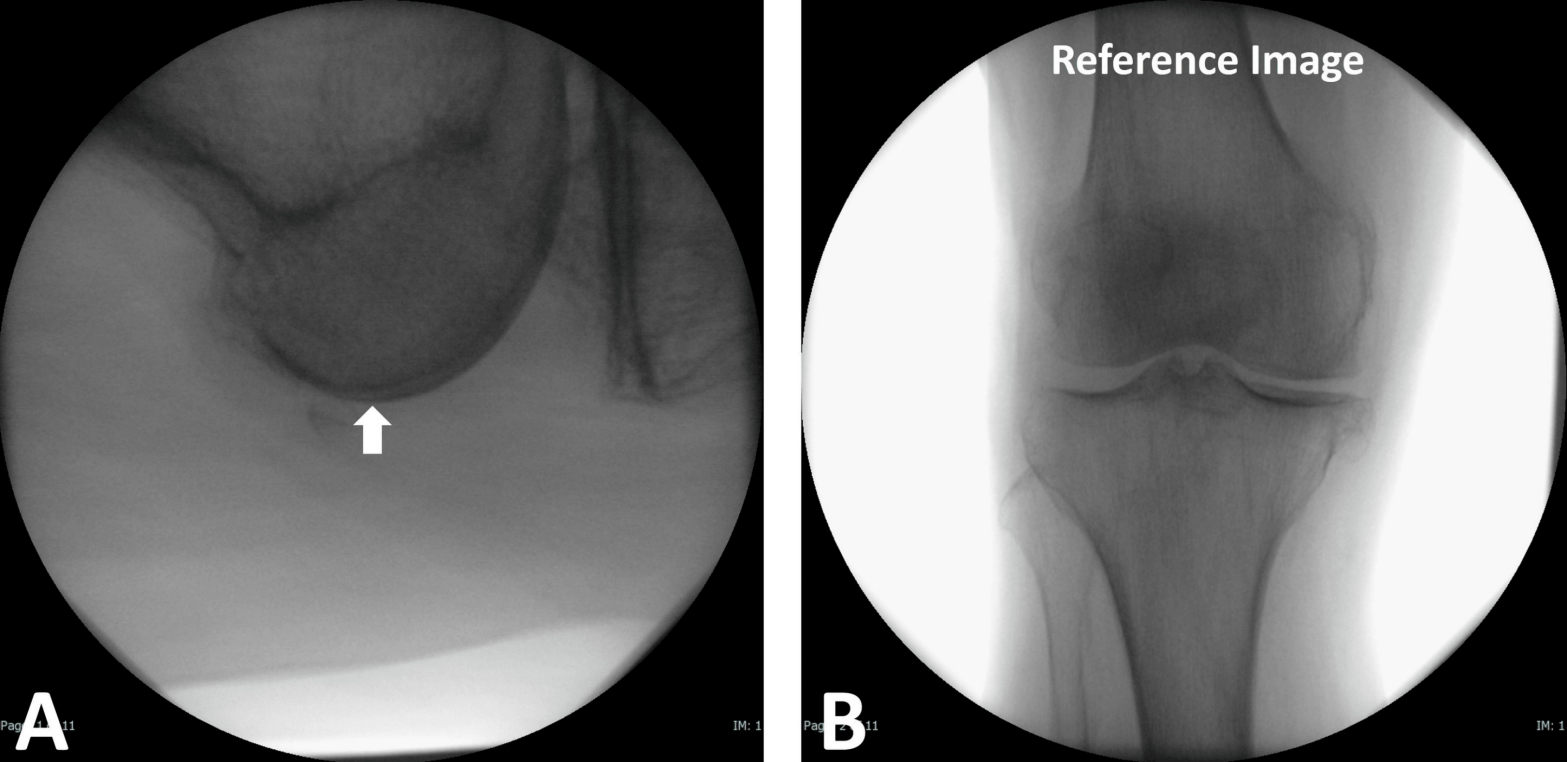
Knee view in the reference position The horizontal lateral knee view revealed a perfect overlap of both posterior condyles (white arrow) at the center of the monitor to establish the "reference position" (A). The anterior-posterior knee view at this reference position was saved as the "reference image" (B).

The three-point plane (tabletop plane), based on the posterior edge of the greater trochanter and both posterior condyles, was positioned parallel to the floor in the scissors position, with the lower limb pulled on a traction table (ALPHAMAXX, MAQUET, Rastatt, Baden-Württemberg, Germany) (Figure 1). The parallel alignment in the sagittal plane was confirmed via palpation and visual inspection. The parallel alignment in the axial plane was achieved by ensuring a perfect overlap of both posterior condyles in the horizontal lateral knee view (Figure 2A). This position was defined as the "reference position." The anterior-posterior knee view at the reference position was saved as the "reference image" (Figure 2B). The scissors position was then changed to the surgical position (contralateral to the frog-leg position).

Measuring anteversion during surgery

**Figure 3:**
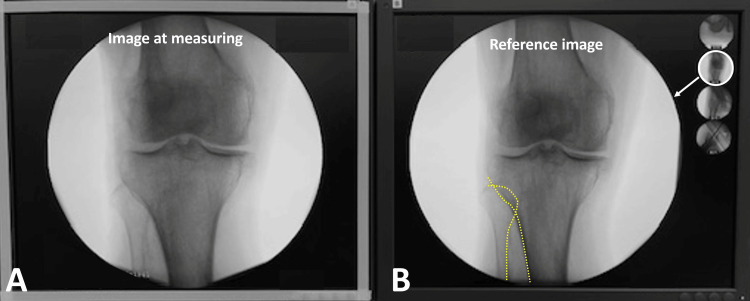
Knee image matching with reference image The reference position for measuring the angle of anteversion was re-established by matching the knee image with the reference image (A). The similarity between both images was assessed by comparing the outlines (dashed line) of the fibula and tibia (B).

**Figure 4:**
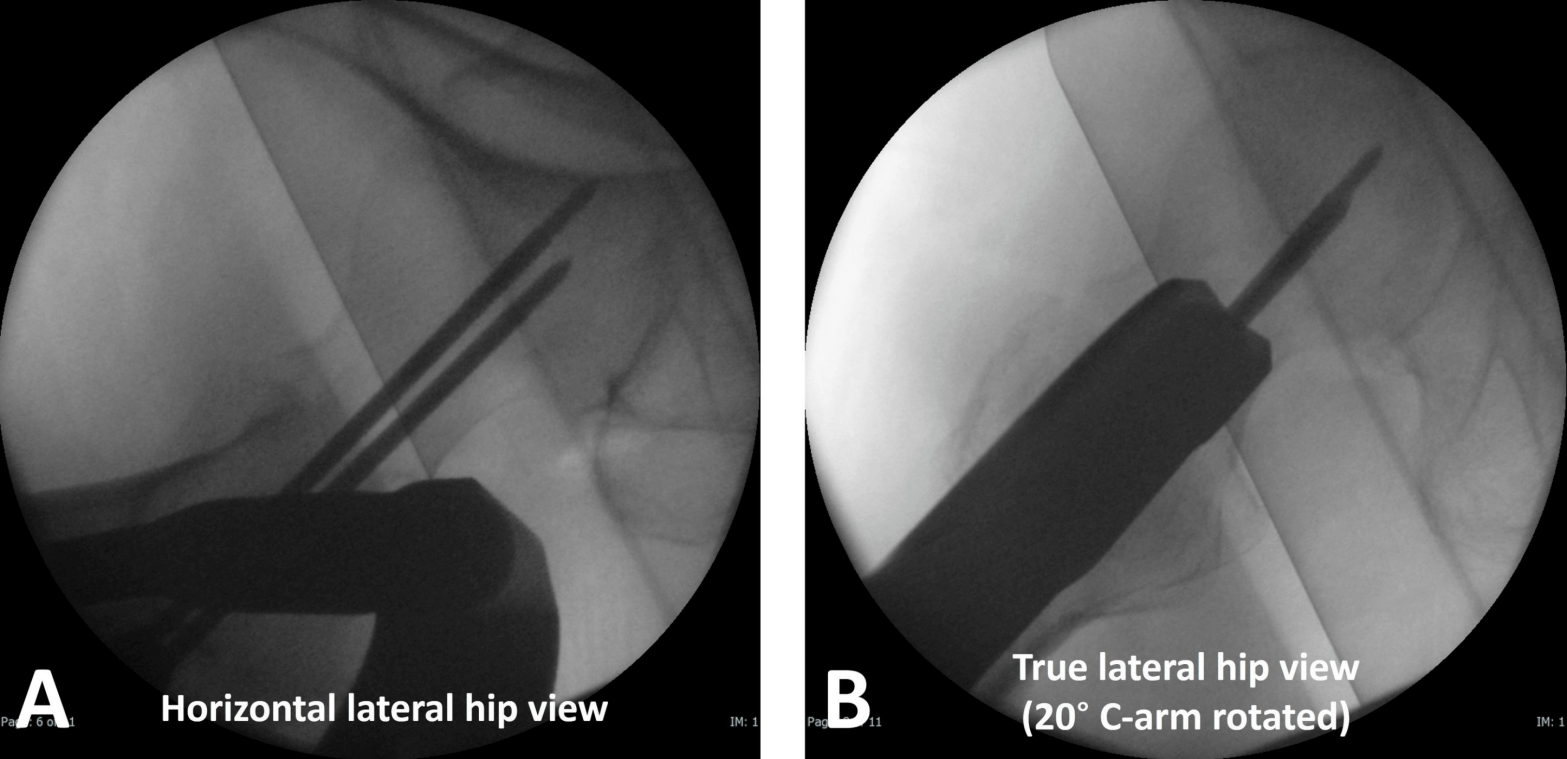
Saving the “measuring image” The horizontal lateral hip view, which included the full head and neck contour, was saved as a "measuring image" (A). If the horizontal lateral hip view did not capture complete head and neck contours, the true lateral hip view was selected as the measuring image. This true lateral hip view (B) was obtained by rotating the C-arm 20° from the horizontal lateral hip view (A).

After inserting the lag screw guide pin, the reference position was re-established using the anterior-posterior knee view matching the reference image (Figure 3) on a fluoroscopic monitor (SIREMOBIL Compact L; Siemens, Munich, Bavaria, Germany). The horizontal lateral hip view, including the full head and neck contour, was then saved as the "measuring image" (Figure 4A). If the horizontal lateral hip view did not include the full head and neck contours, the true lateral hip view was selected as the measuring image (Figure 4B).

**Figure 5:**
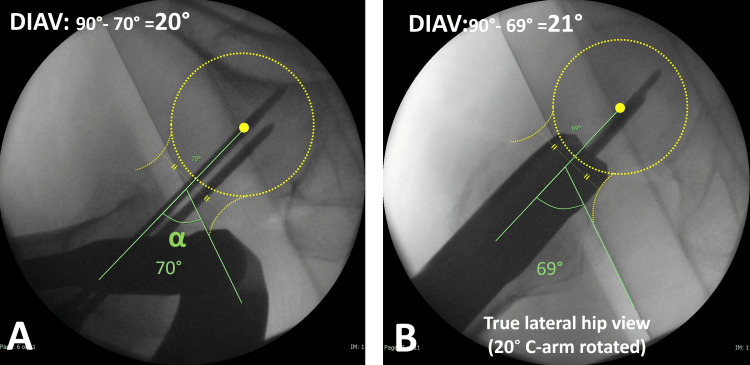
Measuring direct intraoperative anteversion (DIAV) The angle “α” between the neck axis and the hip post axis was measured using the digital tool in the imaging software (A). The neck axis was defined as the line connecting the center of the femoral head to the neck bisector. The hip post axis was represented by the contour of the hip post, positioned vertical to the floor. DIAV was calculated as “90° minus α”. The DIAV measured from the true lateral hip view was similar to that obtained from the horizontal lateral hip view (B).

On the measuring image, the angle difference between the neck axis and the vertical line to the contour of the hip post was measured using the digital tool of the imaging software, Synapse Vincent Volume Analyzer (FUJIFILM, Tokyo, Japan), as the direct intraoperative anteversion (DIAV) (Figure 5). The surgeons changed their sterile gloves and measured the angles on the monitor. The DIAV was recorded in the surgical report, after which the lag screw was inserted.

Eight orthopedic surgeons (four residents and four expert doctors) measured the DIAV during surgery according to the protocol. The key images (Figure 2A, Figure 2B, Figure 3A, and Figure 4A or Figure 4B) were assessed for their acceptability in measuring anteversion. Patients lacking at least one of these acceptable key images and those with previous fractures or prostheses in the ipsilateral femur were excluded from the study.

Evaluation after surgery

**Figure 6:**
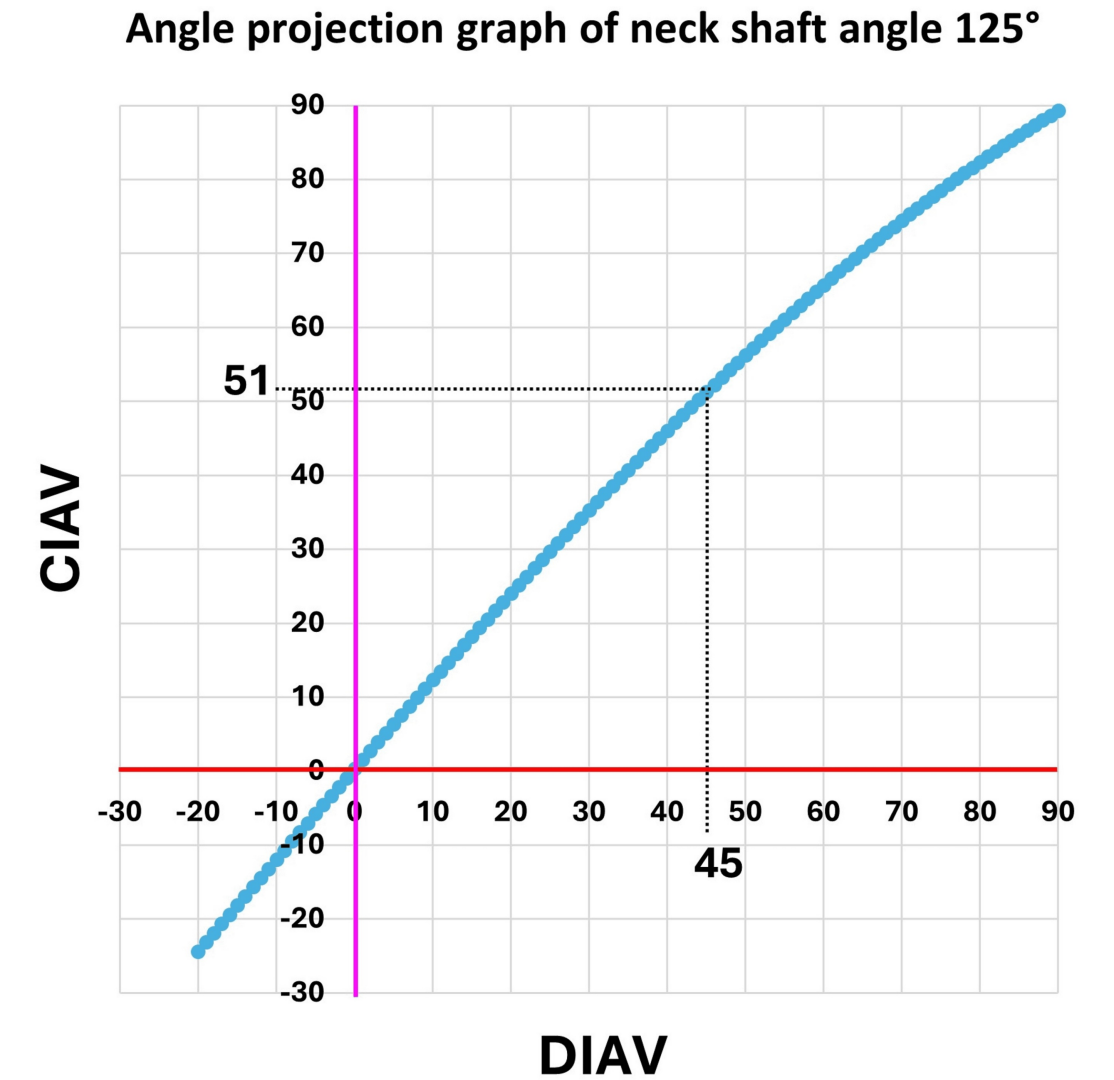
Angle projection graph of neck shaft angle 125° Direct intraoperative anteversion (DIAV) was converted to corrected intraoperative anteversion (CIAV). This conversion graph was based on an axial projection of the lag screw anteversion of the nail with a neck shaft angle of 125°. For example, a DIAV of 45° was corrected to a CIAV of 51°.

DIAV was converted to corrected intraoperative anteversion (CIAV) using a quick chart (Table 1) derived from the graph for angle correction to the axial projection at a neck shaft angle of 125° (Figure 6). The plot points of the graph were determined from a simulation analysis using three-dimensional computer graphics (3DCG) in our previous study [14].

**Table 1:** Quick chart for correction Axial projected correction for normal anteversion range derived from the angle projection graph of neck-shaft angle 125° (Figure 6). DIAV: direct intraoperative anteversion; CIAV: corrected intraoperative anteversion For example, a DIAV of 17° was corrected to a CIAV of 20°.

DIAV	-14° to -10°	-9° to -5°	-4° to 4°	5° to 9°	10° to 14°	15° to 19°	20° to 24°	25° to 34°	35° to 45°
Axial projected correction	add -2°	add -1°	add 0°	add 1°	add 2°	add 3°	add 4°	add 5°	add 6°
CIAV	-16° to -12°	-10° to -6°	-4° to 4°	6° to 10°	12° to 16°	18° to 22°	24° to 28°	30° to 39°	41° to 51°

**Figure 7:**
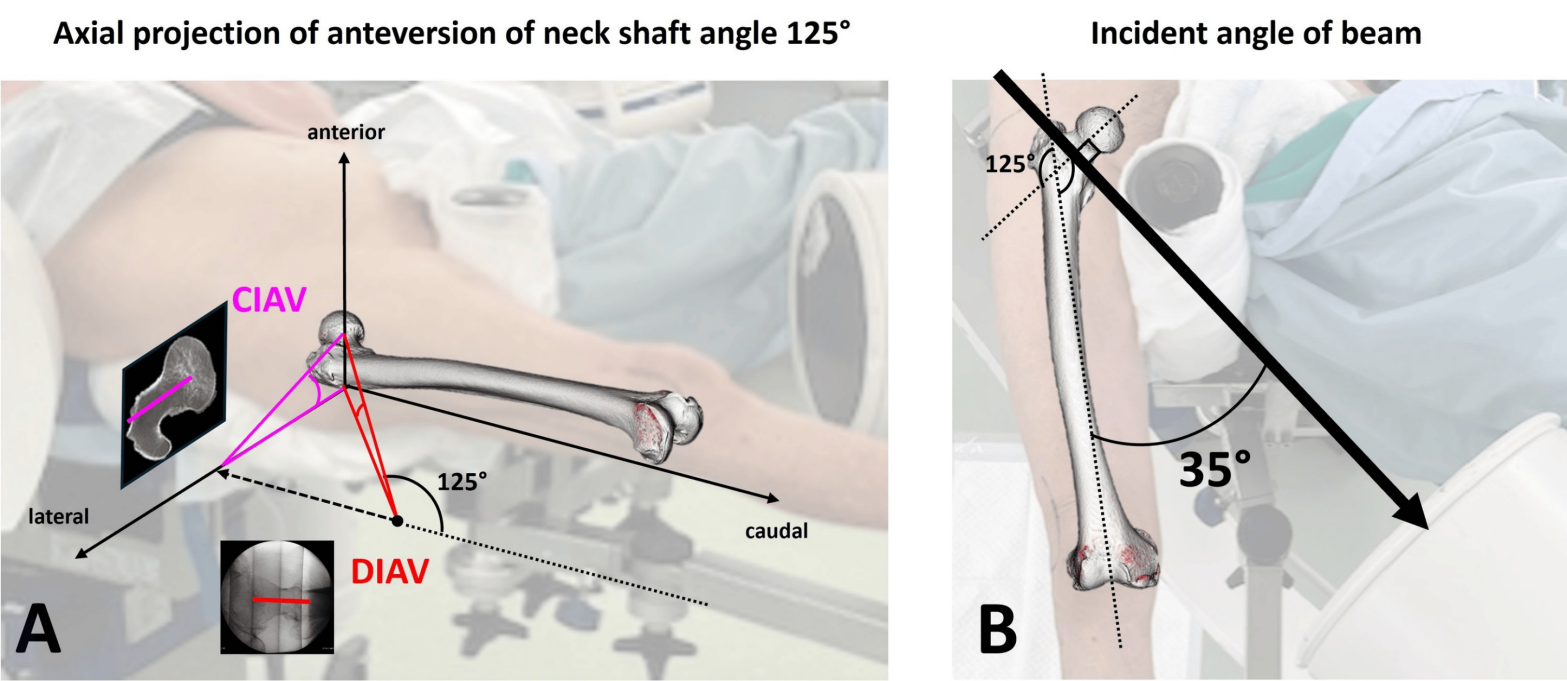
Axial projection of anteversion on horizontal lateral hip view Direct intraoperative anteversion (DIAV) was converted into corrected intraoperative anteversion (CIAV). The CIAV refers to the value measured in the same plane as the axial section of the CT image (A). The condition of a neck shaft angle of 125° indicates that the beam of the image intensifier is set at an incident angle of 35° to the femoral diaphyseal axis and vertical to the neck axis, based on a femur with a neck shaft angle of 125° (B).

CIAV represents the value measured in the same plane as the axial section of the CT image (Figure 7A). The condition of a neck shaft angle of 125° indicated that the beam of the image intensifier was set at an incident angle of 35° to the femoral diaphyseal axis and vertical to the neck axis, based on the tabletop-plane coordinate (Figure 7B).

**Figure 8:**
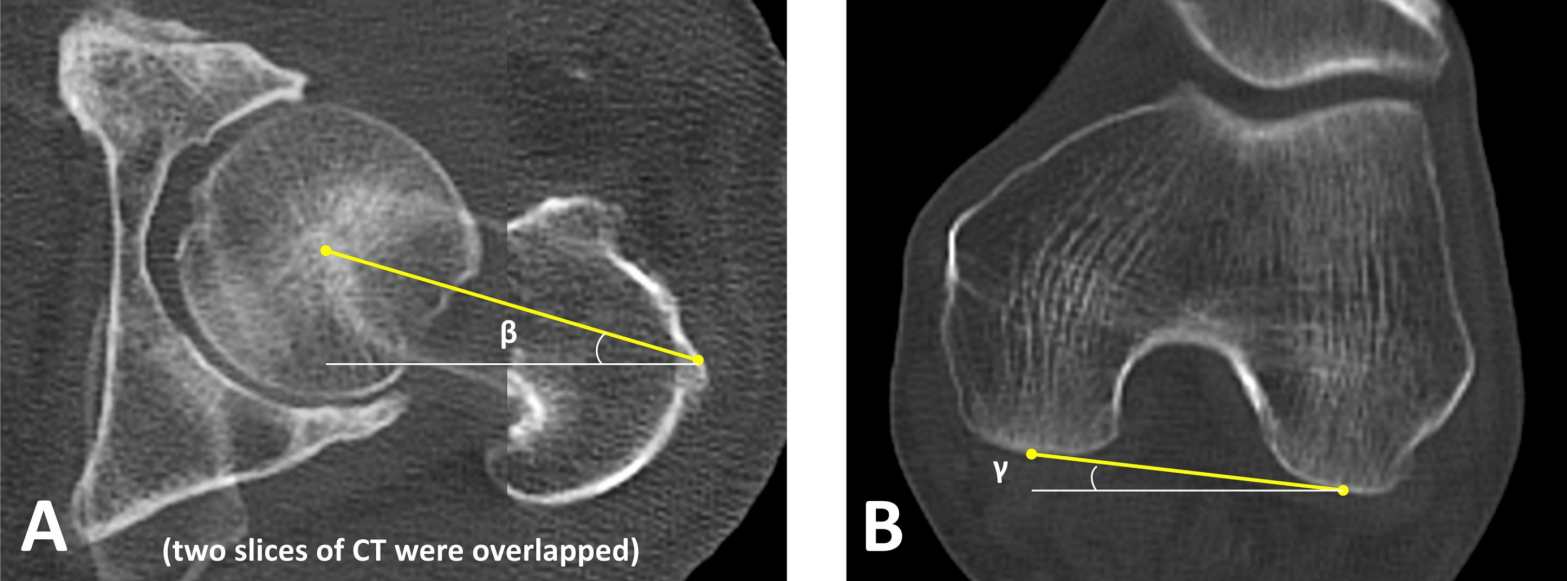
Computed tomography anteversion Computed tomography anteversion (CTAV) was measured as the angle formed between the line intersecting the femoral neck axis and the posterior condylar line of the distal femur. The femoral neck axis was defined as the line connecting the center of the femoral head to the neck bisector on two CT slices where the widest parts of the femoral head and neck were evident (A). The posterior condylar line of the distal femur is defined as the line connecting both edges of the posterior condyles (B). CTAV was calculated as “β minus γ ” in this case.

One week after surgery, a whole femur CT scan was performed using a CT system (SOMATOM Force; Siemens, Munich, Germany). One expert surgeon measured the CT anteversion (CTAV) of the fractured femur on the axial section of the CT using a Synapse Vincent Volume Analyzer. The CTAV was measured as the angle formed between the line intersecting the femoral neck and the posterior condylar line of the distal femur (Figure 8). The femoral neck axis was defined as the line between the center of the femoral head and neck bisector in two CT slices where the widest parts of the femoral head and neck were evident [16].

Statistical analysis

Descriptive statistics were reported as follows. The angle measurements were tested for normality using the Shapiro-Wilk test, with all variables following a normal distribution: DIAV (W = 0.9911, p = 0.7514), CIAV (W = 0.9885, p = 0.5466), and CTAV (W = 0.9877, p = 0.4834). The mean and standard deviation of the angle measurements as well as the mean, median, and range of differences between DIAV or CIAV and CTAV were calculated. To evaluate the accuracy between DIAV or CIAV and CTAV, the Pearson correlation coefficient, Bland-Altman plot, and paired t-test were employed. Statistical analyses were conducted using the EZR analysis software v1.5 (The R Foundation for Statistical Computing, Vienna, Austria, https://www.R-project.org/) [17] and Modified R Commander v4.0.2 (https://personal.hs.hirosaki-u.ac.jp/pteiki/research/stat/R/).

Result

A total of 100 patients (25 male, 75 female) were included in the study. The mean age was 86.7 years (range: 67-104 years). According to the Orthopaedic Trauma Association classification, there were 66 patients classified as A1, 23 as A2, and 11 as A3. The displacement of the proximal fragment was classified as intramedullary in 36 patients, extramedullary in 51, and anatomical in 13. The mean DIAV and CIAV were 8.2°±8.2° and 9.6°±9.7°, respectively, while the mean CTAV was 10.3°±10.4°.

**Figure 9:**
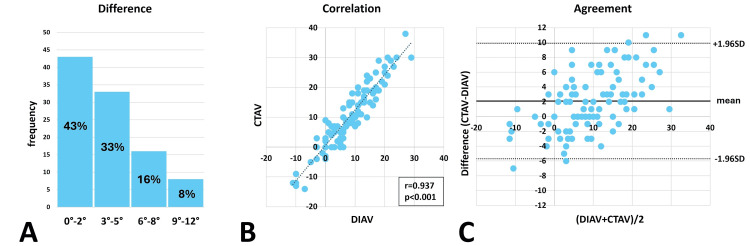
Difference, correlation, and agreement, between DIAV and CTAV Histogram of the difference between direct intraoperative anteversion (DIAV) and computed tomography anteversion (CTAV) (the median difference between DIAV and CTAV is 3.0° (range: 0°–11°), with 76 patients exhibiting a difference of ≤5°) (A). Correlation analysis revealed a Pearson’s correlation coefficient of r = 0.937 (p < 0.001) (B). The Bland–Altman plot indicates agreement (SD: standard deviation); 96 patients fell within the limits of agreement, while the plot distribution exhibited a proportional trend and an upward offset (the limits of agreement are between +1.96SD and -1.96SD) (C).

The mean difference between DIAV and CTAV was 3.5±2.8°. The median difference was 3° (range: 0°-11°), with 76 patients exhibiting differences of ≤5°. DIAV was significantly lower than CTAV (p < 0.001, paired t-test), and the two measurements were strongly correlated (Pearson’s correlation coefficient r = 0.937, p < 0.001). The Bland-Altman plot revealed that 96 patients were within the limits of agreement, while the plot distribution exhibited a proportional trend and an upward offset (Figure 9).

**Figure 10:**
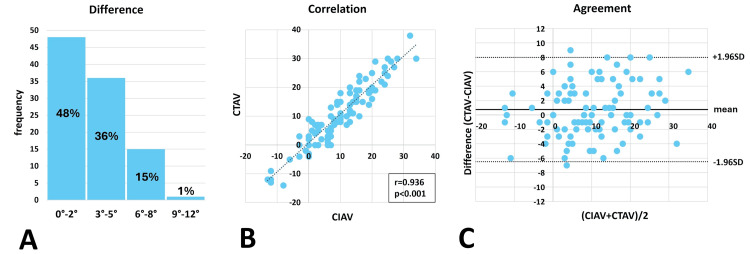
Difference, correlation, and agreement, between CIAV and CTAV Histogram of the difference between corrected intraoperative anteversion (CIAV) and computed tomography anteversion (CTAV) (the median difference between CIAV and CTAV is 3.0° (range: 0°–9°), with 84 patients exhibiting a difference of ≤5°(A). Correlation analysis revealed a Pearson’s correlation coefficient of r = 0.937 (p < 0.001) (B). The Bland–Altman plot indicates agreement (SD: standard deviation); 98 patients fell within the limits of agreement, and the plot distribution exhibited no trend (the limits of agreement are between +1.96SD and -1.96SD) (C).

The mean difference between CIAV and CTAV was 3.0°± 2.2°. The median difference was 3° (range: 0°-9°), with 84 patients exhibiting differences of ≤5°. No significant differences were found between the CIAV and CTAV (p = 0.054, paired t-test), and the two measurements were strongly correlated (Pearson correlation coefficient r = 0.936, p < 0.001). The Bland-Altman plot revealed that 98 patients were within the limits of agreement, and the plot distribution showed no trend (Figure 10).

## Discussion

This is the first study to directly measure femoral anteversion during trochanteric fracture surgery. CIAV demonstrated sufficient accuracy in estimating CTAV.

Summary of results

The Pearson correlation coefficient between DIAV and CTAV was similar to that between CIAV and CTAV. This result suggests that the accuracy of DIAV has the potential to be comparable to that of CIAV.

The distribution of >95% within the limits of agreement on the Bland-Altman plot indicates high agreement between the two measurements, shown not only between CIAV and CTAV but also between DIAV and CIAV. However, the Bland-Altman plot distribution between DIAV and CTAV exhibited a proportional trend with an offset of 2.0° upward from zero. In contrast, the distribution of the Bland-Altman plot between CIAV and CTAV showed no such trend, with an offset of only 0.75° upward from zero. The smaller offset from zero indicates a lesser error between the two measurements, whereas a proportional trend suggests a systemic error between them. The Bland-Altman plot demonstrated a higher agreement for CIAV than for DIAV.

The paired t-test revealed no significant differences between CIAV and CTAV, whereas significant differences were observed between DIAV and CTAV. This result suggests that the CIAV can be used intraoperatively to estimate measurements similar to those of the CTAV.

The mean difference between CIAV and CTAV was 3.0°± 2.2°, and the median difference was 3° (range: 0°-9°), with 84 patients exhibiting differences of ≤5°. These results demonstrate sufficient accuracy of CIAV as an indicator to prevent malrotation >15°.

Assessment of measurement method

The measurement of femoral anteversion is affected by the neck shaft angle and limb position [18]; therefore, a proper setting of the coordinate system for measurement is necessary. This method utilizes the anteversion angle shown between the neck axis and the vertical line of the hip post axis in the femoral neck lateral view, provided that the tabletop plane is positioned parallel to the floor and the hip post axis is vertical to the floor.

Patients lacking at least one of the acceptable key images (Figure 2A, Figure 2B, Figure 3A, and Figure 4A or Figure 4B) were excluded from this study because the accuracy of setting the coordinates depended on these key images. Specifically, the matching of the anterior-posterior knee view with the “reference image” was not accurate in several patients. The similarity between these two images was assessed by comparing the outlines of the fibula and tibia (Figure 3). Achieving a match with the reference image required a similar tilt and positioning of the knee on the monitor as in the reference image, because visual judgment can be challenging on anteroposterior knee views. The lateral view of the knee was more accurate than the anteroposterior knee view for establishing a reference image. However, a lateral knee view cannot be obtained via fluoroscopy with the patient in the surgical position (contralateral frog-leg position). Switching to the scissors position during surgery consumes time and labor while maintaining a sterile environment.

The hip post is often used on a traction table during surgery for trochanteric fractures, positioned vertically to the floor, and displayed in the lateral hip views. Therefore, measuring anteversion based on the hip post axis offers benefits in terms of cost and universality. If the contour of the hip post is unclear, attaching a thin metal rod vertically to the hip post can help clarify the axis. In this study, the angle of anteversion was measured using a digital tool in the imaging software; however, it was also possible to measure the angle of the lateral hip view using an analog protractor.

This measurement method increased the operative time by approximately five to 10 minutes and the fluoroscopic time by a few minutes, due to preparation before surgery and measurements during surgery, respectively. However, we believe that the benefits of anteversion measurement outweigh the drawbacks of extended time. The cost associated with this measurement method does not increase compared to the cost without it.

The measurement of femoral anteversion on two-dimensional (2D) CT shows the axial projection of the three-dimensional (3D) femoral anteversion (Figure 7A). DIAV closely reflects the 3D femoral anteversion. Axial projection correction is necessary to compare DIAV with the 2DCT anteversion after surgery. In this study, axial projection correction was calculated based on the correction graph for a neck-shaft angle of 125°. A neck-shaft angle of 125° indicated that the image intensifier beam was set at an incident angle of 35° to the femoral diaphyseal axis and vertical to the neck axis (Figure 7B). However, the same incident angle could not always be set. In fact, an incident angle between 25° and 45° was likely used during surgery. Despite this variation, the systemic error observed in the Bland-Altman plot between DIAV and CTAV was corrected in the plot between CIAV and CTAV. Moreover, in our previous study [14], 3DCG simulation estimated that axial projection graphs were nearly identical in models with neck-shaft angles of 125°, 130°, and 135°. The axial projection correction method used in this study was sufficiently effective to achieve agreement in the measurements.

Additional suggestion

This method of measuring anteversion during surgery for femoral trochanteric fractures can also be applied for femoral diaphyseal fractures. Furthermore, the measurements of anteversion in diaphyseal fractures may be more accurate than those in trochanteric fractures because of the absence of damage to the neck trochanter region. Additionally, a “reference image” is not required, as the reference position can be set precisely and easily by achieving perfect overlap of both posterior condyles in the horizontal lateral knee view while in the scissors position during surgery.

Limitation

The error in the reference position: The tabletop plane is affected by the displaced greater trochanteric fragment and relies on palpation and visual judgment.

The error in matching with the reference image: The judgment of the matching anteroposterior knee view with the reference image depends solely on visual assessment. This error directly affects anteversion measurements.

The error in the measurement of DIAV: The measurement of the angle between the neck axis and the hip post axis may include interobserver and intraobserver errors. The measured images were the horizontal lateral hip view or the true lateral hip view, which were not the same. However, the error was acceptable (Figure 5).

The error in axial projection: Axial projection correction was calculated based on the correction graph for a neck shaft angle of 125°. The same conditions cannot be set each time.

The error in the angle measurement of CT: CTAV was measured twice by one surgeon, which may have introduced an inter-observer error. CTAV might be affected by limb position at CT scanning.

Next step

This method evaluates femoral anteversion before the insertion of the lag screw, allowing for adjustments if the anteversion is deemed unacceptable. In the next study, during surgery for trochanteric fractures, patients whose femoral anteversion difference was reduced to <15° from that of a normal femur using this method will be compared to patients without evaluation of anteversion. This may clarify whether this method contributes to the prevention of malrotation and the improvement of functional outcomes.

## Conclusions

CIAV showed statistically significant agreement with CTAV. This study has potential limitations, such as reliance on a single expert for CTAV measurements and a limited sample size. However, the measurement method for assessing femoral anteversion using the hip post axis on the lateral hip image with the tabletop plane parallel to the floor during surgery demonstrated sufficient accuracy to indicate anteversion in femoral trochanteric fractures.
